# Pseudoexfoliation Syndrome and Antidepressant Drug Use

**DOI:** 10.4274/tjo.galenos.2018.06887

**Published:** 2019-02-28

**Authors:** Erdoğan Yaşar, Nilgün Yıldırım, Eray Atalay

**Affiliations:** 1 Aksaray University Training and Research Hospital, Ophthalmology Clinic, Aksaray, Turkey; 2 Eskişehir Osmangazi University Faculty of Medicine, Department of Ophthalmology, Eskişehir, Turkey

**Keywords:** Pseudoexfoliation, depression, antidepressant drug

## Abstract

**Objectives:**

To investigate the relationship between pseudoexfoliation syndrome (PES) and the use of antidepressant medications.

**Materials and Methods:**

This population-based, cross-sectional study included 2,017 of 2,356 invited subjects who were randomly selected from the Turkish Statistical Institute database (www.turksat.gov.tr/) as part of an epidemiologic study which specifically aimed to detect the prevalence of PES in the province of Eskişehir. During the examination, a detailed questionnaire was administered to determine physician-diagnosed systemic disease and drug use.

**Results:**

Eight of the 2,017 participants in the study were excluded for various reasons (2 with posterior synechiae, 2 with corneal opacity, 1 uncooperative patient, 1 eviscerated patient, 1 with active adenoviral keratoconjunctivitis, and 1 with a history of angle closure). PES was detected in 100 (5%) of the 2,009 patients whose data were analyzed. The percentage of subjects with concurrent antidepressant drug use in the PES and non-PES non-glaucoma groups were 4.1% (n=3) and 1.1% (n=21), respectively. The difference between the two groups was statistically significant (p=0.024). In addition, the use of medications for hypertension (p<0.001) and coronary artery disease (p=0.009) was also higher in the PES group.

**Conclusion:**

The higher prevalence of antidepressant drug use in patients with PES may be related to the processes of vascular damage and inflammation common to the pathogenesis of both PES and depression, as well as the high rate of chronic systemic comorbidities in these patients.

## Introduction

Pseudoexfoliation syndrome (PES), first described by Lindberg in 1917, is a clinical entity characterized by the accumulation of gray-white extracellular fibrillary material in the anterior segment tissues of the eye.^1,2^ Besides the eye, exfoliative material (EM) has also been detected in the heart, lung, liver, kidney, and meninges using light/electron microscopy and immunohistochemical/biochemical methods.^3,4^

Oxidative damage and inflammation caused by free radicals has been shown to have a role in the pathogenesis of PES.^5,6,7^ Increased oxidative stress and subsequent impairment of cellular immunity through the proteasome system are believed to be instrumental in the pathogenesis of PES.^1^ Electron microscope studies of iris tissue samples from patients with PES have demonstrated EM deposition and damage to the iris vessels.^8,9^ Furthermore, histopathological examination of samples obtained from PES patients with aortic aneurysm revealed focal accumulation of EM, pronounced fibrosis, and tunica intima elastosis in the adventitial and subendothelial connective tissue.^10^ A recent genetic study identified five novel loci associated with predisposition for PES and the risk ratio for one of these loci varied by geographical latitude (increasing toward the polar regions).^11^

PES is a clinical condition that increases in frequency in individuals over the age 50 and with increasing age.^12^Various studies have shown that the incidence and prevalence of PES varies in different populations, even within the same population in different regions, and its frequency varies between 0% and 38%.^13^

In addition to secondary glaucoma, cataract, and complications related to cataract surgery, PES patients have higher rates of hypertension (HT), coronary artery disease (CAD), heart attack, stroke, Alzheimer’s disease, peripheral vascular diseases, and hearing loss, illustrating the systemic character of PES.^14,15,16,17,18,19,20^ In addition, a study comparing patients with pseudoexfoliation glaucoma (PXG), primary open-angle glaucoma (POAG), and a control group showed that depression was significantly more common in the pseudoexfoliative group while the POAG and control groups had similar rates of depression, suggesting that PES may be associated with depression.^21^ The aim of this study was to investigate the relationship between PES and depression and other systemic diseases based on survey results from our population-based cross-sectional PES prevalence study.

## Materials and Methods

The study was carried out in accordance with the principles of the Declaration of Helsinki and ethical approval was obtained from the Ethics Committee of Eskişehir Osmangazi University (7 February 2013, project number: 06). This population-based randomized study was part of an epidemiological study to determine the prevalence of PES in Eskişehir, Turkey, a city covering 2,678 km^2^ with a population of 826,716.^22^ Randomization was done with a centralized method using the Turkish Statistical Institute database. This database contains regularly updated address information for all residents of Eskişehir. The target population consisted of people aged 40 and over living in the urban center and rural areas of Eskişehir.

A layered two-stage cluster sampling method was used for randomization and the study was conducted between 15 June and 1 October 2014 using the most recent information from February 2014. In the first sampling phase, clusters of approximately 100 households were probability sampled in proportion to their size. In the second phase, 10 households were randomly selected from each cluster using systemic sampling. Residence data for the selected households were obtained from local archives. People aged 40 years and over who resided in the randomly selected households were contacted through the neighborhood representative and invited to participate in the study. Brochures explaining the purpose of the study and information about the disease were given to neighborhood representatives to deliver to these individuals. Examinations were conducted between June and October 2014 and informed consent was obtained from all participants.

The subjects were examined in the ophthalmology outpatient clinic of the Eskişehir Osmangazi University School of Medicine. Data pertaining to the participants’ demographic and social characteristics, general and ocular medical histories, and regular drug use were collected in face-to-face interviews by a nurse who was knowledgeable about the study and survey methods. Participants were questioned about all drugs they were currently using. Anterior segment examination was performed by an experienced resident physician (E.Y.) using a photobiomicroscope (Topcon-sl-D7, SN:1613331, Japan). Intraocular pressure was measured with an Icare tonometer; the average of 5 measurements provided by the device was recorded for both eyes. In terms of glaucoma, all patients underwent fundus examination (cup-to-disc [C/D] ratio) and Humphrey visual field test when necessary for diagnosis. Glaucomatous optic neuropathy was defined as neuroretinal rim loss with vertical C/D ratio ≥0.7 or vertical C/D ratio asymmetry >0.2 between the two eyes and/or visual field defect typical of glaucoma and consistent with focal notching of the neuroretinal rim. Both pupils were then dilated using mydriatic drops (0.1% tropicamide). Thirty minutes after instillation, the lens and other anterior segment structures were re-evaluated for pseudoexfoliation and photographed. PES diagnosis was made in the presence of white-gray EM on the pupil margin and lens anterior capsule. Images of patients with diagnosed or suspected PES were evaluated by the glaucoma consultant for confirmation (N.Y.).

### Statistical Analysis

All statistical analyses were performed using SPSS version 21.0 (SPSS, Inc., Chicago, IL). A t-test was used to compare numerical variables and chi-square test was used to compare the distribution of categorical variables between groups. Statistical significance was accepted as p<0.05.

## Results

Of the 2,356 people who were randomly selected and invited to the study, 2,017 (85.6%) participated. Eight of the 2,017 participants (2 with posterior synechiae, 2 with corneal opacities, and 4 who were uncooperative, had one eviscerated eye, had adenoviral conjunctivitis, or had history of narrow-angle glaucoma) were excluded from the analysis. Demographic characteristics of the individuals with and without PES who were included in the study are summarized in Table 1.

PES was detected in 100 (5%) of the 2,009 participants. The mean age of the 100 people with PES was 69.1±9.9 years and that of the individuals without PES was 59.2±10.9 years (p<0.001).

After 33 glaucoma patients were excluded from 1,909 non-PES subjects, antidepressant drug use was reported by 21 (1.1%) of the remaining 1,876 subjects (mean IOP: 14.7±3.4 mmHg). After 26 glaucoma patients were excluded from 100 subjects with PES, antidepressant drug use was reported by 3 (4.1%) of the remaining 26 subjects (mean IOP: 14.1±3.3 mmHg). There was no statistical difference in mean IOP between the groups, whereas the incidence of antidepressant use was significantly higher in patients with PES (p=0.024, Table 2). Details regarding the physician-prescribed antidepressant drugs are shown in Table 3.

In addition, the results of the survey questions regarding physician-diagnosed disease and drug use revealed that of the 1,909 non-PES subjects, 626 (32.8%) used antihypertensive drugs and 162 (8.5%) used medication for CAD. These rates were significantly higher in the 100 subjects with PES, with 48% using antihypertensive drugs (p<0.001) and 17% taking medication for CAD (p=0.009). No significant difference was found between the groups in terms of drugs used for other diseases (p>0.05) (Table 4).

## Discussion

In our study, PES was detected in 100 of 2,009 individuals evaluated (5%). Other studies of PES prevalence in the Turkish population reported rates in the 7-12% range, higher compared to our population-based randomized study.^23,24,25,26^ This may be explained by the fact that those studies were hospital-based.

Antidepressant drug use was identified in 21 (1.1%) of 1,876 people without PES or glaucoma and in 3 (4.1%) of 74 patients with PES but without glaucoma, which was a statistically significant difference (p=0.024).

There is only one study in the literature showing an association between PES and depression, and it was conducted in patients with PXG. Cumurcu et al.^21^ evaluated the prevalence of depression in 41 PXG patients, 32 POAG patients, and 40 control groups. Based on findings from other researchers indicating that depression was common in chronic disease and reasoning that glaucoma was a chronic disease, they expected depression rates to be higher in both PXG and POAG patients; however, only the PXG group showed a significantly higher prevalence of depression compared to the control group.^27^ The higher prevalence of depression in the PXG group suggested that the vascular damage involved in the pathogenesis of depression might be associated with pseudoexfoliation, supporting the vascular depression hypothesis.^27,28^ Unlike the study by Cumurcu et al.,^21^ we excluded patients with glaucoma to include subjects with PES only. Numerous studies have shown that depression is more common in patients with CAD, diabetes, and HT.^29,30^ Of the 6 participants with PES and psychiatric disease identified in our study, 4 had concomitant diabetes, HT, or CAD; considering the vascular etiology of depression, this may have increased antidepressant use in these patients. The presence of multiple chronic comorbidities may have led to depression in these individuals.

Oxidative damage and inflammation caused by free radicals was shown to have a role in the pathogenesis of PES.^5,6,7^ Various studies have also reported that oxidative stress and inflammation may be involved in the pathogenesis of depression.^31,32^ Inflammation induced by the accumulation of pseudoexfoliative material in the brain may trigger depression and increase antidepressant drug use in PES patients.

Moreover, ischemic heart disease, history of angioplasty, HT, and hearing loss were more common in the PES group than in controls. The higher prevalence of other diseases, even if not statistically significant, may be a factor contributing to antidepressant drug use. Loss of health and the limitations imposed by chronic diseases cause the higher incidence of depression with these diseases.^33^ Various studies have shown that depression is more common in patients with CAD, diabetes, asthma, and cancer.^34,35,36,37^

Of the 100 PES patients in our study, 48% had HT and 17% had ischemic heart disease; consistent with the literature, these rates were significantly higher when compared with patients without PES (p<0.001). Previous studies have demonstrated the relationship between PES and systemic diseases such as HT, CAD, heart attack, peripheral vascular diseases, ischemic brain diseases, stroke, and Alzheimer’s disease.^14,15,16,17,18,19,20^ In a recent study, 62% of 260 patients who presented for cataract surgery had cardiovascular diseases, 46.5% with HT and 19.7% with ischemic heart disease.^38^ Furthermore, a significant relationship has been reported between PES and sensorineural hearing loss.^39,40^

### Study Limitations

Limitations of our study are that we did not perform brain imaging in PES patients using antidepressant drugs to rule out a central etiology of depression, and we did not evaluate for family history of depression.

## Conclusion

The higher prevalence of antidepressant drug use among patients with PES illustrates the need for further research to determine the relationship between PES and depression.

## Figures and Tables

**Table 1:**
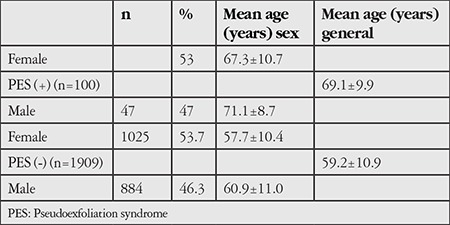
Age and sex distribution of participants with and without pseudoexfoliation syndrome

**Table 2:**
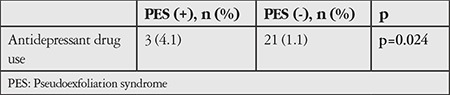
Comparison of antidepressant use in participants with and without pseudoexfoliation syndrome

**Table 3:**
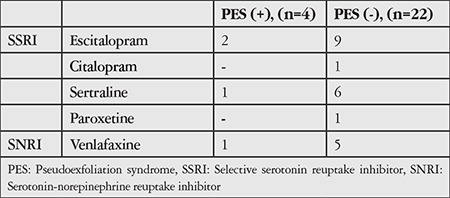
The distribution of participants with and without pseudoexfoliation syndrome diagnosed with depression and using antidepressant drugs

**Table 4:**
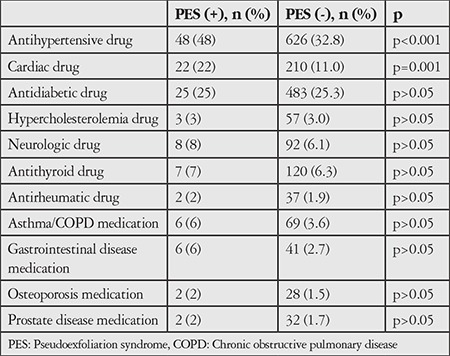
Comparison of systemic drug use in participants with and without pseudoexfoliation syndrome
